# Collaboration in Facial Plastic Surgery: “Tis Not in Numbers but in Unity That Our Great Strength Lies,” *Thomas Paine, Common Sense (1776)*

**DOI:** 10.1089/fpsam.2021.0185

**Published:** 2021-09-03

**Authors:** Anthony P. Sclafani, Alwyn D'Souza, Travis T. Tollefson

**Affiliations:** ^1^ Editors-in-Chief, Facial Plastic Surgery.; ^2^ Department of Otolaryngology—Head & Neck Surgery, Weill Cornell Medicine, New York, New York, USA.; ^3^ Department of ENT, University Hospital Lewisham, London, England, United Kingdom.; ^4^ Editor-in-Chief, Facial Plastic Surgery & Aesthetic Medicine.; ^5^ Department of Otolaryngology—Head and Neck Surgery, University of California, Davis, Sacramento, California, USA.

## Facial Plastic Surgery

The journal *Facial Plastic Surgery* was founded in the early 1980s by two titans of the facial plastic and reconstructive surgical world—Gene Tardy and Tony Bull. These two friends and giants of our field sought to span the Atlantic and create the first journal solely dedicated to advancing knowledge in facial plastic surgery. The journal would select leaders in the field to serve as guest editors of “themed” issues, who would then commission top surgeons to write “state-of-the-art” review or expert opinion articles within that theme. Led by editors from both sides of the Atlantic and published by Thieme Medical Publishers, the journal sought both authors and an audience international in nature. In recognition of the rapidity with which research in the field was growing, *Facial Plastic Surgery* began accepting free papers on topics important to the facial plastic surgery community in 2010; the journal has published original research from all continents except Antarctica. In this new model, *Facial Plastic Surgery* now publishes all accepted articles (commissioned and “unsolicited” papers) online as soon as possible. The editors and publisher have committed to a rapid review of all submissions and e-publication of all articles within 8 weeks of acceptance. *Facial Plastic Surgery* is the official journal of the European Academy of Facial Plastic Surgery, and while known to the U.S. facial plastic surgery community, we welcome the opportunity to work more closely with members of the American Academy of Facial Plastic and Reconstructive Surgery (AAFPRS) and the editorial team of *Facial Plastic Surgery & Aesthetic Medicine*.

## Facial Plastic Surgery & Aesthetic Medicine

Facial plastic surgery has become an international specialty with outstanding global contributions. In that spirit, we look forward to a new collaboration with an old friend. Our field is blessed to have two journals with different backgrounds and strengths, unified in a commitment to further the art and science of facial plastic surgery. Wayne Larrabee launched *Archives of Facial Plastic Surgery* 21 years ago and then, *JAMA Facial Plastic Surgery* under editor, John Rhee. The journal recently transitioned to the American Academy of Facial Plastic and Reconstructive Surgery (AAFPRS) with a new name *Facial Plastic Surgery & Aesthetic Medicine* and an impact factor of 3.78. This journal name represents the surgical and non-surgical diversity of facial plastic surgeons' practices as the official journal of the AAFPRS. The journal is also affiliated with the European Academy of Facial Plastic Surgery, The Australian Academy of Facial Plastic Surgery, and the International Federation of Facial Plastic Surgery Societies. The editorial team, AAFPRS, and new publisher (Mary Ann Liebert, Inc.) intend to build upon this rich history as a multi-specialty, peer-reviewed journal that seeks to enhance patient outcomes by providing accurate and innovative original research.

We look forward to a collegial and productive collaboration between these two journals as our international specialty grows. These two journals have a shared history and commitment to excellence for our readers. We recognize that our specialty of facial plastic surgery will productively grow throughout the world by publishing both rigorous scientific studies and expert opinions, contributing to the consolidation of the knowledge and attitudes of our field. Readers should find both journals online with subscription access to *Facial Plastic Surgery* at https://www.thieme.in/facial-plastic-surgery-journal and *Facial Plastic Surgery & Aesthetic Medicine* (http://www.liebertpub.com/fpsam).

In Solidarity for Our Specialty, the Editors:

Anthony P. Sclafani, MD, MBA

Alwyn D, Souza, MBBS, FRCS

*Facial Plastic Surgery*

Travis T. Tollefson, MD, MPH

*Facial Plastic Surgery & Aesthetic Medicine*

